# Performance of shear-waves elastography in the non-invasive assessment of liver fibrosis in chronic hepatitis in the Romanian population

**DOI:** 10.1186/1471-2334-14-S7-O15

**Published:** 2014-10-15

**Authors:** Monica Andreea Stoica, Anca Streinu-Cercel, Oana Săndulescu, Liliana Lucia Preoțescu, Gabriela Ceapraga, Adrian Streinu-Cercel

**Affiliations:** 1 National Institute for Infectious Diseases "Prof. Dr. Matei Balş", Bucharest, Romania; 2 Carol Davila University of Medicine and Pharmacy, Bucharest, Romania

## Background

Liver fibrosis is one of the major factors associated with progression of liver disease in chronic HBV [1] or HCV [2,3] infection, but also in metabolic diseases with impact on the liver.

## Methods

We have performed a study to determine liver stiffness in patients with chronic hepatitis in Romania. One trained operator performed shear-waves elastography (SWE) using Aixplorer (SuperSonic Imagine, Aix-en-Provence, France) in all consecutive patients monitored in our clinic over the course of 7 months, from January 2014 to July 2014.

## Results

We have examined a total of 80 patients with chronic hepatitis, of which 58.8% had HCV infection, 16.3% HBV infection, 6.3% HBV+HDV coinfection, 2.5% ASH, 2.5% HIV infection and 13.8% had idiopathic liver involvement. The male-to-female ratio was 0.86:1, and the mean age was 48.6±14.9 years.

The mean duration of hepatic disease evolution was 7.6±5.7 years, longer for HCV infection (mean 8.3±5.9 years) than for HBV infection (4.75±3.9 years, p=0.028). The overall mean SWE liver stiffness was 9.6±5.3 kPa, higher in patients with HCV infection (10.8±5.9 kPa) than in those with HBV infection (6.98±1.9 kPa, p=0.009). Overall, 37.5% of patients were classified as F0-F1 on SWE, 25.0% F2, 8.8% F3 and 28.7% F4.

Liver cirrhosis was present in 28.7% of patients and hepatocellular carcinoma had already been diagnosed in 6.3% of all patients and in 21.7% of all patients with cirrhosis (5 cases, of which 4 had been previously diagnosed with cirrhosis with HCV – 3 cases, and HBV+HDV – 1 case, and 1 had an idiopathic cause for liver involvement and a stiffness corresponding to F0-F1 on SWE).

## Conclusion

There seem to be significant differences between two of the main groups of patients examined, with a longer duration of infection and an accordingly higher liver stiffness in the chronic HCV group, when compared to the chronic HBV group.
